# Antiepileptic Drugs Elevate Astrocytic Kir4.1 Expression in the Rat Limbic Region

**DOI:** 10.3389/fphar.2018.00845

**Published:** 2018-08-06

**Authors:** Takahiro Mukai, Masato Kinboshi, Yuki Nagao, Saki Shimizu, Asuka Ono, Yoshihisa Sakagami, Aoi Okuda, Megumi Fujimoto, Hidefumi Ito, Akio Ikeda, Yukihiro Ohno

**Affiliations:** ^1^Department of Pharmacology, Osaka University of Pharmaceutical Sciences, Takatsuki, Japan; ^2^Department of Neurology, Wakayama Medical University, Wakayama, Japan; ^3^Department of Epilepsy, Movement Disorders and Physiology, Graduate School of Medicine, Kyoto University, Kyoto, Japan

**Keywords:** astrocytes, Kir4.1 channels, epilepsy, antiepileptic drugs, amygdala

## Abstract

Inwardly rectifying potassium (Kir) channel subunits Kir4.1 are specifically expressed in astrocytes and regulate neuronal excitability by mediating spatial potassium buffering. In addition, it is now known that astrocytic Kir4.1 channels are closely involved in the pathogenesis of epilepsy. Here, to explore the role of Kir4.1 channels in the treatment of epilepsy, we evaluated the effects of the antiepileptic drugs, valproate, phenytoin, phenobarbital and ethosuximide, on Kir4.1 expression in astrocytes using immunohistochemical techniques. Repeated treatment of rats with valproate (30–300 mg/kg, i.p., for 1–10 days) significantly elevated the Kir4.1 expression levels in the cerebral cortex, amygdala and hippocampus. Up-regulation of Kir4.1 expression by valproate occurred in a dose- and treatment period-related manner, and did not accompany an increase in the number of astrocytes probed by glial fibrillary acidic protein (GFAP). In addition, repeated treatment with phenytoin (30 mg/kg, i.p., for 10 days) or phenobarbital (30 mg/kg, i.p., for 10 days) also elevated Kir4.1 expression region-specifically in the amygdala. However, ethosuximide (100 mg/kg, i.p., for 10 days), which can alleviate absence but not convulsive seizures, showed no effects on the astrocytic Kir4.1 expression. The present results demonstrated for the first time that the antiepileptic drugs effective for convulsive seizures (valproate, phenytoin, and phenobarbital) commonly elevate the astrocytic Kir4.1 channel expression in the limbic regions, which may be related to their antiepileptic actions.

## Introduction

Epilepsy is a chronic neurologic disease characterized by recurrent convulsive and/or non-convulsive seizures, affecting approximately 70 million people worldwide (nearly 1% of the population) (Banerjee et al., 2009; Ngugi et al., 2010; Zack and Kobau, 2017). Various antiepileptic drugs, which predominantly act on the neuronal ion channels (e.g., blockers of voltage-gated Na^+^ and Ca^2+^ channels) and the inhibitory GABAergic system (e.g., stimulants of GABA_A_ receptor/Cl^−^ channel complex and inhibitors of GABA transaminase), are currently used in the treatment of epilepsy (Meldrum and Rogawski, 2007). Therapy with these standard antiepileptic drugs provides adequate control in about 70% of epilepsy patients; however, the remaining 30% of patients still suffer from refractory (treatment-resistant) symptoms and are sometimes subjected to surgical treatments (e.g., ablation of seizure foci, deep brain stimulation and vagus nerve stimulation) (Mattson, 1998).

Inwardly rectifying potassium (Kir) channel subunits Kir4.1 are specifically expressed in the brain astrocytes and form Kir4.1-containing channels (Kir4.1 channels), homo-tetramers of Kir4.1 subunits and hetero-tetramers of Kir4.1 and Kir5.1 subunits (Neusch et al., 2006; Ohno et al., 2015; Ohno, 2018). Kir4.1 channels play a key role in mediating the spatial potassium buffering currents, which remove excessive extracellular potassium ions (K^+^) at tripartite synapses (Walz, 2000; Kofuji and Newman, 2004; Simard and Nedergaard, 2004; Ohno et al., 2015). Namely, neurons release a considerable amount of K^+^ during the repolarization process of action potentials, elevating the local extracellular K^+^ concentration (e.g., about 1 mM per spike) at synapses. Kir4.1 channels in astrocytes conduct inward potassium currents and transport K^+^ to sites of lower concentrations such as microvessels. In addition, Kir4.1 channels regulate the resting membrane potential of astrocytes, which serves as a driving force of astrocytic glutamate uptake through excitatory amino-acid transporter 2 (EAAT2) (Olsen and Sontheimer, 2008; Frizzo, 2017). Therefore, dysfunction of Kir4.1 channels elevates not only extracellular K^+^, but also glutamate levels at tripartite synapses (Ohno et al., 2015; Ohno, 2018). Furthermore, we recently demonstrated that inhibition (channel blockade or expressional suppression) of Kir4.1 channels facilitated the expression of brain-derived neurotrophic factor (BDNF) in astrocytes, which may produce diverse effects including synaptic plasticity, neural sprouting, neurogenesis and reactive gliosis in the brain (Kinboshi et al., 2017a; Ohno, 2018).

It is now known that Kir4.1 plays an important role in inducing and developing epilepsy (epileptogenesis). Kir4.1 knockout mice showed severe motor impairment (e.g., ataxia and tremor), epileptic symptoms (e.g., jerky movements and convulsive seizures), and early mortality (Kofuji et al., 2000; Neusch et al., 2001; Djukic et al., 2007). In addition, astrocytic Kir4.1 expression was reported to be reduced (down-regulated) in the brain regions related to seizure foci in patients with epilepsy and animal models of epilepsy (Ferraro et al., 2004; Inyushin et al., 2010; Das et al., 2012; Heuser et al., 2012; Steinhäuser et al., 2012; Harada et al., 2013). Furthermore, it has been shown that loss-of-function mutations (i.e., missense and nonsense mutations) in the human *KCNJ10* gene encoding Kir4.1 caused the epileptic disorders known as “EAST/SeSAME” syndrome (Bockenhauer et al., 2009; Scholl et al., 2009; Reichold et al., 2010). Patients with EAST/SeSAME syndrome manifested generalized tonic-clonic seizures (GTCSs) within a few months after birth, in addition to sensorineural deafness, ataxia and electrolyte imbalance. Therefore, it is likely that Kir4.1 channels are closely involved in the pathogenesis of epilepsy. However, the roles of Kir4.1 channels in the treatment of epilepsy or the influences of antiepileptic drugs on Kir4.1 expression are still unknown.

Besides the acute neural inhibition, repeated treatments with antiepileptics are known to exert, to some extent, prophylactic effects in chronic epilepsy, although the underlying mechanisms remain unclear (Iudice and Murri, 2000; Michelucci, 2006; Torbic et al., 2013). This makes a hypothesis that antiepileptics may enhance Kir4.1 expression to prevent epileptogenesis. In the present study, therefore, we evaluated the effects of the antiepileptic drugs, valproate, phenytoin, phenobarbital and ethosuximide, on astrocytic Kir4.1 expression to explore the potential role of Kir4.1 expression in the treatment of epilepsy.

## Materials and methods

### Animals

Male 6-week-old SD rats (Japan SLC, Shizuoka, Japan) were used. The animals were kept in air-conditioned rooms (24 ± 2°C and 50 ± 10% relative humidity) under a 12-h light/dark cycle (light on: 8:00 a.m.) and allowed *ad libitum* access to food and water. The animal care methods complied with the Guide for the Care and Use of Laboratory Animals of the Ministry of Education, Science, Sports and Culture of Japan. The experimental protocols of this study were approved by the Animal Research Committee of Osaka University of Pharmaceutical Sciences.

### Drug treatments and brain sampling

Animals (6 rats/group) were intraperitoneally injected with a daily dose of an antiepileptic drug as followed; valproate (30, 100, and 300 mg/kg), phenytoin (30 mg/kg), phenobarbital (30 mg/kg), or ethosuximide (100 mg/kg) for 10 days. To evaluate the time-course, animals were treated with valproate (300 mg/kg) for 1 or 5 day(s). The test doses of each drug were set to anticonvulsive doses in rodents, according to previous papers (Walton and Treiman, 1989; Lothman et al., 1991; Löscher, 1999; Gören and Onat, 2007). Twenty-four hours after the last drug treatment, the animals were deeply anesthetized with pentobarbital (80 mg/kg, i.p.), transcardially perfused with ice-cold phosphate-buffered saline (PBS) and then with 4% paraformaldehyde solution. The brains were then removed from the skull and placed in fresh fixative for at least 24 h.

### Immunohistochemical analysis

Expression of Kir4.1 and glial fibrillary acidic protein (GFAP: a specific marker for astrocytes) in each brain region were analyzed by immunohistochemical staining using the avidin-biotin complex (ABC) method, as published previously (Harada et al., 2013; Nagao et al., 2013). After blocking, sections were incubated with rabbit polyclonal anti-Kir4.1 antibodies (1:100; Alomone Labs, Jerusalem) or mouse monoclonal anti-GFAP antibodies (1:100; Progen, Heidelberg, Germany) at 4°C overnight. Thereafter, they were incubated with biotinylated goat anti-rabbit IgG antibodies (1:400, Vector Laboratories, Burlingame, CA, USA) or biotinylated goat anti-mouse IgG antibodies (1:400, Sigma-Aldrich, St. Louis, MO, USA) for 60 min and with avidin-biotinylated horseradish peroxidase complexes (Vectastain ABC Kit, Vector Laboratories) for an additional 60 min. Kir4.1- and GFAP-immunoreactivity (IR) were visualized by the diaminobenzidine-nickel staining method.

**Figure 1:**
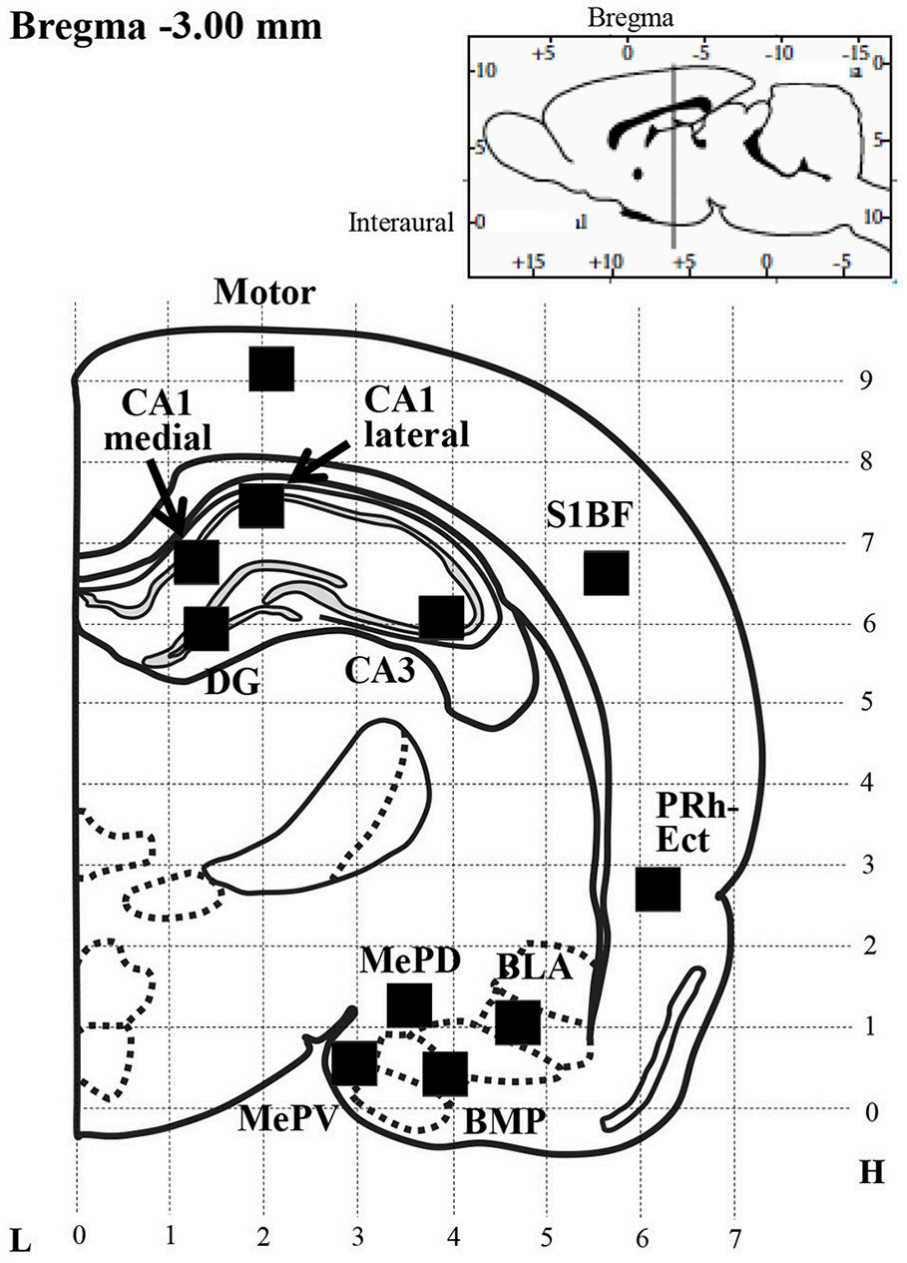
Schematic illustration of a brain section selected for quantitative analysis of immunoreactivity (IR) of Kir4.1 or GFAP. Filled squares in each brain region indicate the areas analyzed for counting of Kir4.1-IR- or GFAP-IR-positive cells, which were set according to the rat brain atlas (Paxinos and Watson, 2007). Motor, motor cortex; S1BF, primary somatosensory cortex barrel field; PRh-Ect, perirhinal-ectorhinal cortex; MePV, medial amygdaloid nucleus posteroventral part; MePD, medial amygdaloid nucleus posterodorsal part; BLA, basolateral amygdaloid nucleus anterior part; BMP, basomedial amygdaloid nucleus posterior part; CA1 medial or lateral, CA3 and DG, hippocampal CA1 medial, CA1 lateral, CA3 and dentate gyrus; L, Lateral coordinates (mm); H, Horizontal coordinates from interaural line (mm).

Kir4.1 and GFAP expression was quantified by counting the number of Kir4.1- or GFAP-IR-positive cells under a blinded condition in a 350 × 350 μm^2^ grid laid over each brain region of successive sections at the Bregma−3.00 mm level, according to the rat brain atlas (Paxinos and Watson, 2007), as described previously (Iha et al., 2017) (Figure 1). Regions of interest included the motor cortex (Motor), primary somatosensory cortex barrel field (S1BF), perirhinal-ectorhinal cortex (PRh-Ect), medial amygdaloid nucleus posteroventral part (MePV), medial amygdaloid nucleus posterodorsal part (MePD), basolateral amygdaloid nucleus anterior part (BLA), basomedial amygdaloid nucleus posterior part (BMP), hippocampal CA1 medial, CA1 lateral, CA3 and dentate gyrus (DG). The relative expression rate of Kir4.1 was defined as a percentage of the number of Kir4.1-IR-positive cells relative to that of GFAP-IR-positive cells obtained from successive sections.

### Immunofluorescence double staining

In some experiments, immunofluorescence double staining of Kir4.1 with GFAP was performed as published previously (Harada et al., 2013; Nagao et al., 2013). Briefly, fixed brain samples were embedded in paraffin and cut into 4 μm thick sections. Sections were autoclaved for 20 min to retrieve the antigen, and blocked with 1% bovine serum albumin (BSA) for 30 min. The sections were incubated with primary antibodies for GFAP (1:50) and Kir4.1 (1:100) at 4°C overnight. Subsequently, they were incubated with secondary antibodies of tetramethylrhodamine-5-(and 6)-isothiocyanate (TRITC; red fluorescence) goat anti-mouse (1:50; Sigma-Aldrich) or fluorescein isothiocyanate (FITC; green fluorescence) goat anti-rabbit (1:50; Sigma-Aldrich), respectively, for visualization. Immunofluorescence images were obtained with a confocal laser scanning microscope (Carl Zeiss Japan, LSM 700 ZEN, Tokyo, Japan).

### Drugs

Sodium valproate, phenytoin, phenobarbital, and ethosuximide were purchased from Sigma-Aldrich. Other common laboratory reagents were also obtained from commercial sources.

### Statistical analysis

All data are expressed as the mean ± S.E.M. Comparisons between two groups were performed by Student's *t*-test. Statistical significance of differences among multiple groups was determined by one-way ANOVA followed by Tukey's *post hoc* test. A *P*-value of less than 0.05 was considered statistically significant.

## Results

### Effects of valproate on astrocytic Kir4.1 expression

**Figure 2:**
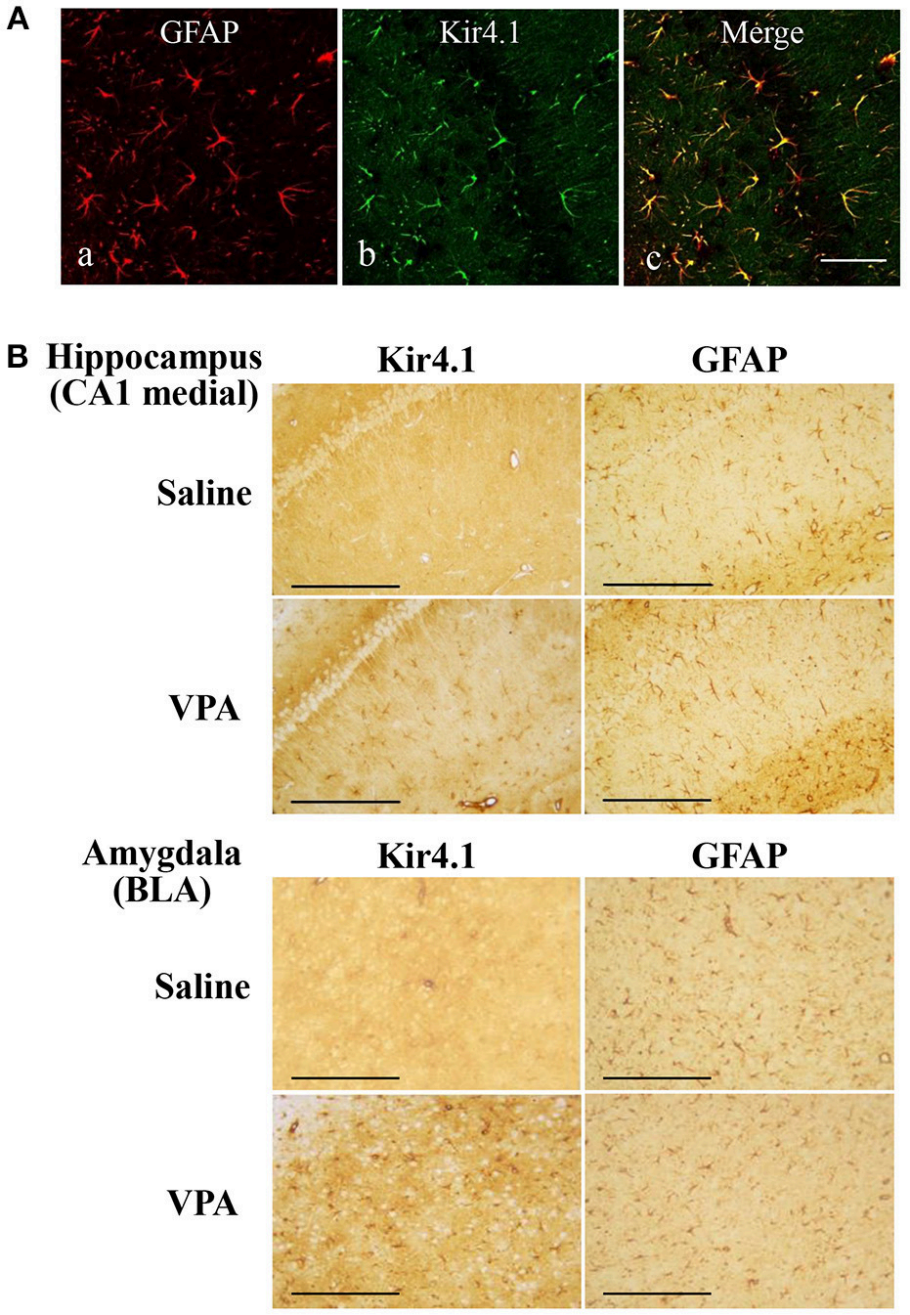
Effects of valproate (VPA) on expression of Kir4.1 and GFAP in the hippocampus. **(A)**: Expressional patterns of Kir4.1 channels in astrocytes. Representative images of immunofluorescence double staining for GFAP and Kir4.1 in a medial part of the hippocampal CA1 region. Scale bar: 50 μm. **(B)** Representative images of immunohistochemical staining for Kir4.1 (left panels) and GFAP (right panels) in the hippocampal CA1 medial regions (medial CA1) and the basolateral amygdaloid nucleus anterior part (BLA) of saline- or valproate (VPA)-treated rats. Scale bar: 200 μm.

We first confirmed the expression pattern of Kir4.1 in rat brains using the immunofluorescence double staining method. As reported previously (Harada et al., 2013), confocal laser microscopic analysis revealed that Kir4.1-IR was specifically expressed in astrocytes (somata and processes of stellate-shaped cells) probed by GFAP (Figure 2A).

**Figure 3:**
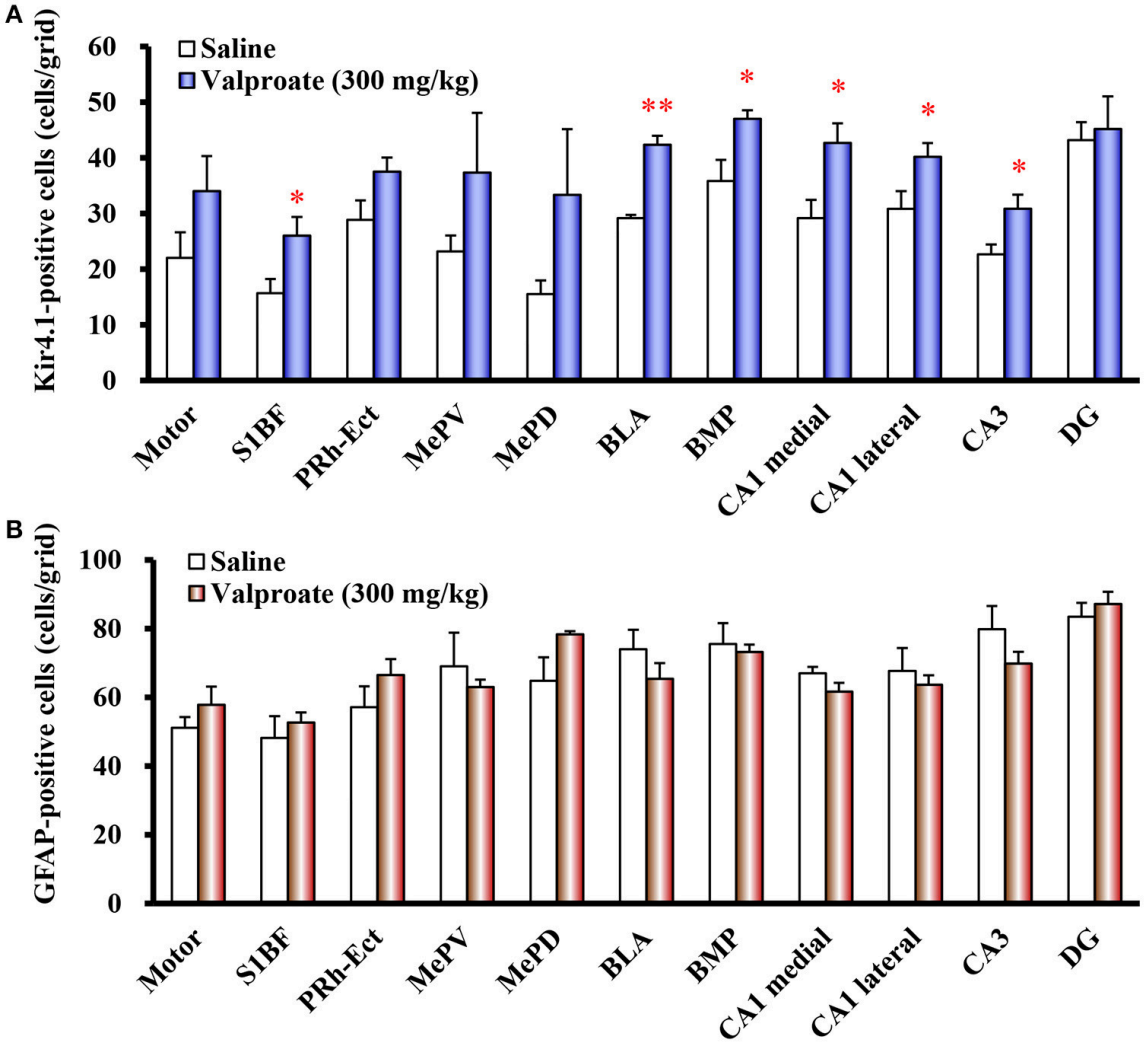
Effects of valproate on topographical expression of Kir4.1 and GFAP in rats. Data show the number of Kir4.1-immunoreactivity (IR) -positive cells **(A)** or GFAP-IR-positive cells **(B)** in each brain region of rats treated with valproate (300 mg/kg/day, i.p.) for 10 days. Each point represents the mean ± S.E.M. of six animals. ^*^P < 0.05, ^**^P < 0.01, significantly different from saline-treated rats.

**Figure 4:**
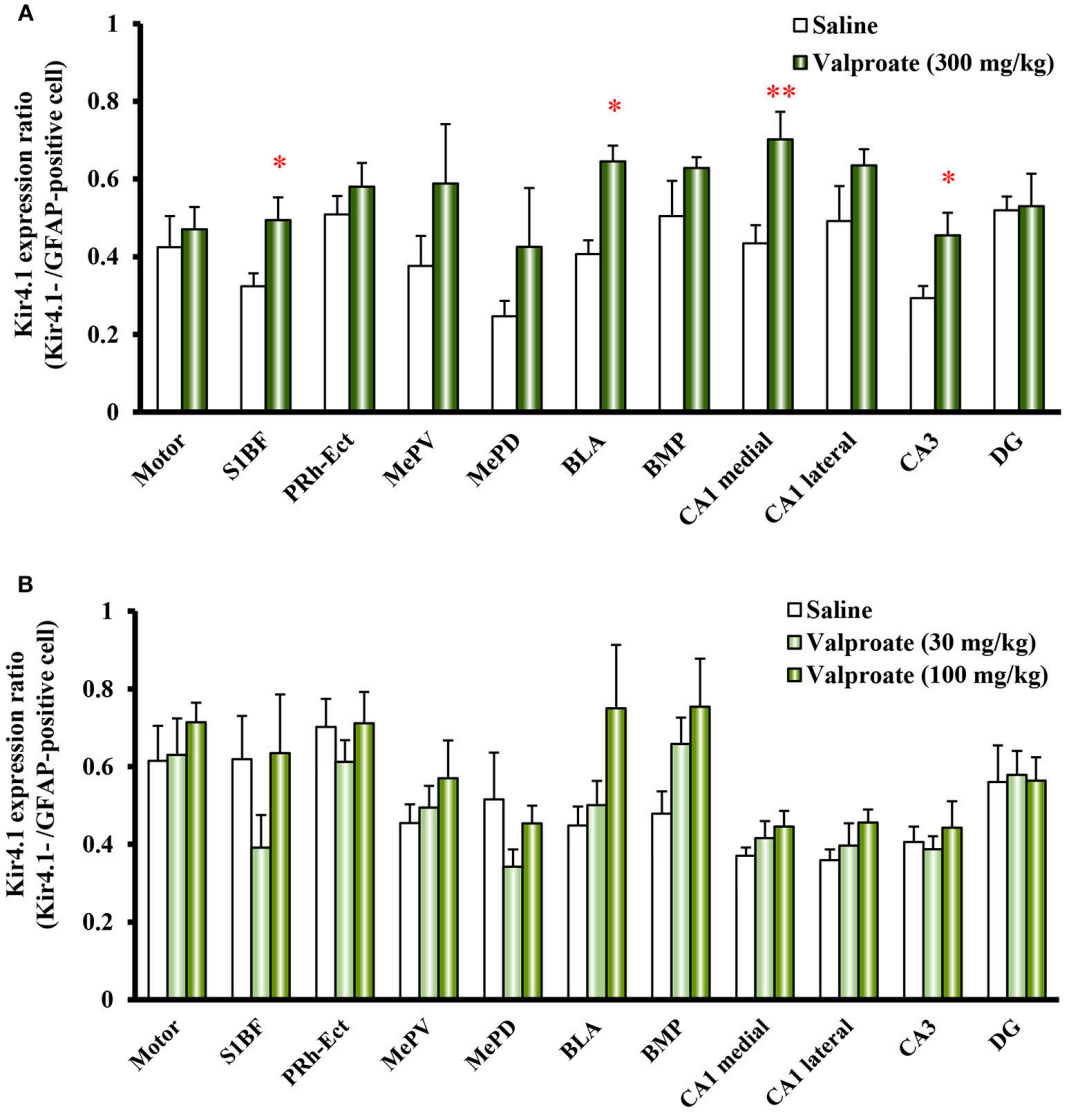
Dose-response effects of valproate on topographical Kir4.1/GFAP expression ratios in rats. Animals were treated with 300 mg/kg/day **(A)** or 30 and 100 mg/kg/day **(B)** of intraperitoneal valproate for 10 days. A pair of successive brain sections was stained with anti-Kir4.1 or anti-GFAP antibodies. The Kir4.1 expression ratios were calculated as the number of Kir4.1-IR-positive cells relative to the number of GFAP-IR-positive cells in each region. Each point represents the mean ± S.E.M. of six animals. ^*^P < 0.05, ^**^P < 0.01, significantly different from saline-treated rats.

**Figure 5:**
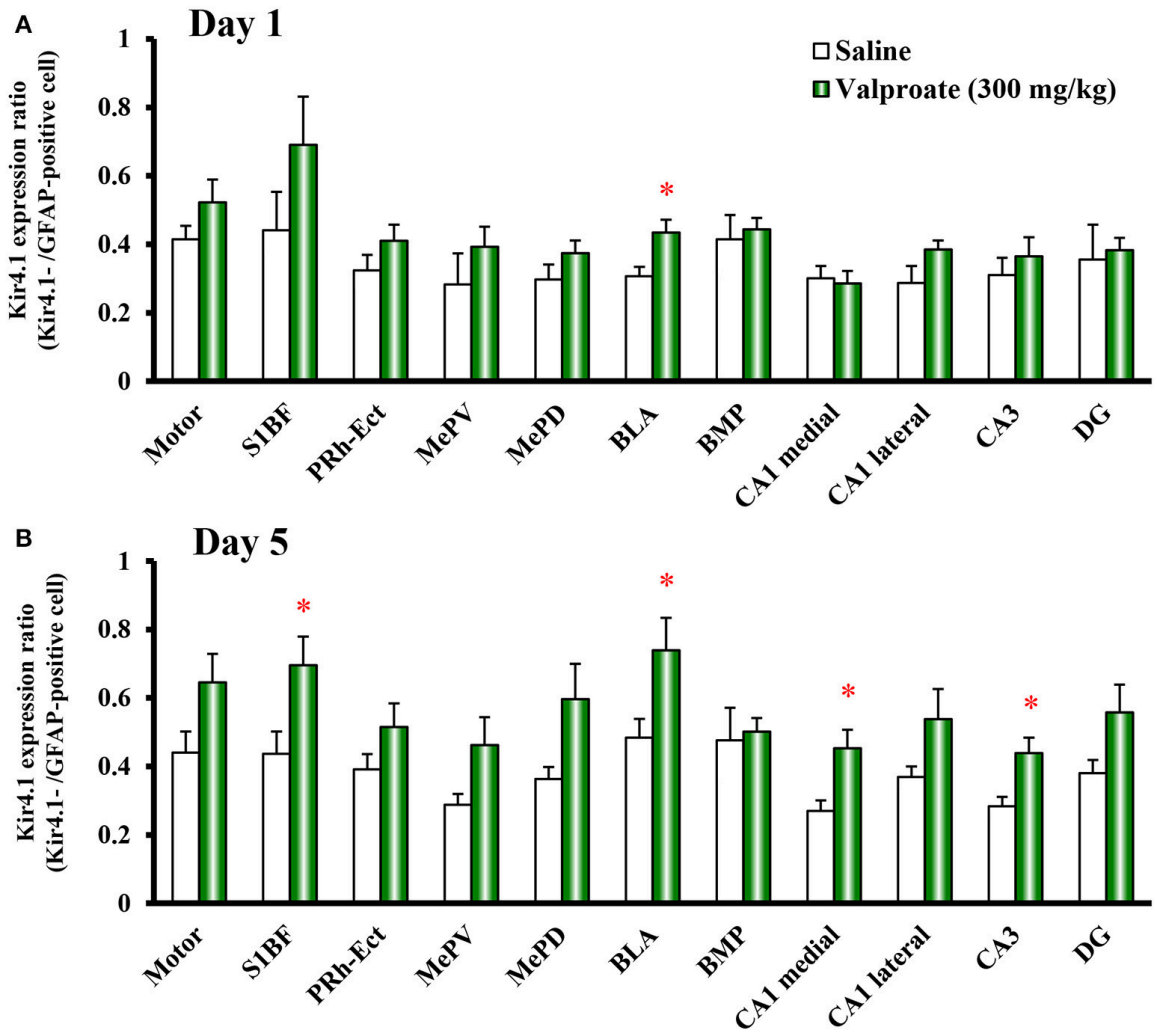
Time-course effects of valproate on topographical Kir4.1/GFAP expression ratios in rats. Animals were treated with 300 mg/kg/day of intraperitoneal valproate for 1 day **(A)** or 5 days **(B)**. The Kir4.1 expression ratios were calculated as the number of Kir4.1-IR-positive cells relative to the number of GFAP-IR-positive cells in each region. Each point represents the mean ± S.E.M. of six animals. ^*^P < 0.05, significantly different from saline-treated rats.

Repeated treatments of animals with valproate (300 mg/kg, i.p.) for 10 days significantly increased the number of Kir4.1-IR-positive astrocytes in the sensory cortex [S1BF: *t*_(10)_ = 2.433, *P* = 0.035], amygdala [BLA: *t*_(10)_ = 7.769, *P* < 0.001 and BMP: *t*_(10)_ = 2.743, *P* = 0.021], and hippocampus [medial CA1: *t*_(10)_ = 2.798, *P* = 0.019, lateral CA1: *t*_(10)_ = 2.324, *P* = 0.042, and CA3: *t*_(10)_ = 2.653, *P* = 0.024] (Figures 2B, 3A). On the other hand, there were no changes in the number of astrocytes (GFAP-IR positive cells) in any brain regions examined (Figures 2B, 3B). We also calculated the Kir4.1 expression ratios relative to the number of astrocytes (Kir4.1-IR positive cells/GFAP-IR positive cells) in successive brain sections obtained from each animal, which also illustrated region-specific increases in Kir4.1 expression in the sensory cortex [S1BF: *t*_(10)_ = 2.530, *P* = 0.030], amygdala [BLA: *t*_(9)_ = 4.4712, *P* = 0.002] and hippocampus [medial CA1: *t*_(10)_ = 3.158, *P* = 0.010 and CA3: *t*_(10)_ = 2.446, *P* = 0.035] (Figure 4A). This increase in Kir4.1 expression by valproate occurred in a dose- and treatment period-dependent manner. Specifically, valproate (30 and 100 mg/kg, i.p. for 10 days) did not significantly affect Kir4.1 expression in any brain regions while it slightly increased Kir4.1 expression in the amygdala (BLA and BMP) (Figure 4B). In addition, the Kir4.1 expression ratios increased according to the treatment period of valproate, in that a single injection of valproate (300 mg/kg) increased Kir4.1 expression only in the basolateral part of the amygdala [*t*_(10)_ = 2.698, *P* = 0.022], extending to the sensory cortex [S1BF: *t*_(8)_ = 2.437, *P* = 0.041] and hippocampus [medial CA1: *t*_(10)_ = 2.922, *P* = 0.015 and CA3: *t*_(10)_ = 2.939, *P* = 0.015] with the 5 days treatments (Figure 5).

### Effects of other antiepileptic drugs on astrocytic Kir4.1 expression

**Figure 6:**
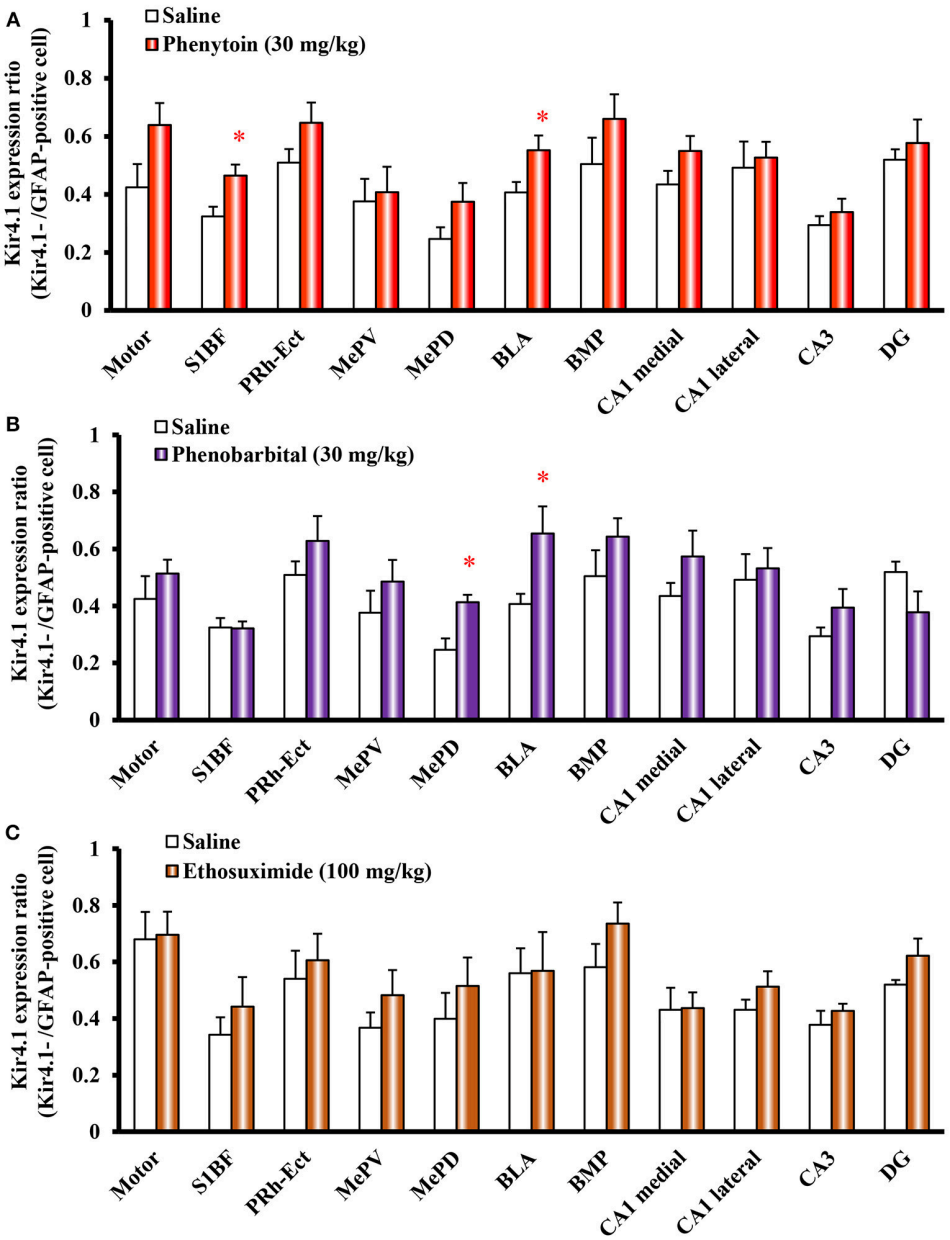
Effects of phenytoin, phenobarbital, and ethosuximide on topographical Kir4.1/GFAP expression ratios in rats. Animals were treated with 30 mg/kg/day (i.p.) of phenytoin **(A)**, 30 mg/kg/day (i.p.) of phenobarbital **(B)** or 100 mg/kg/day (i.p.) of ethosuximide **(C)** for 10 days. The Kir4.1 expression ratios were calculated as the number of Kir4.1-IR-positive cells relative to the number of GFAP-IR-positive cells in each region. Each point represents the mean ± S.E.M. of six animals. ^*^P < 0.05, significantly different from saline-treated rats.

We next examined the effects of other antiepileptic drugs, phenytoin, phenobarbital and ethosuximide, on astrocytic Kir4.1 expression. Repeated treatment of animals with phenytoin (30 mg/kg, i.p.) or phenobarbital (30 mg/kg, i.p.) continuously for 10 days significantly increased the number of Kir4.1-IR-positive astrocytes without affecting the number of astrocytes (GFAP-IR positive cells). The Kir4.1 expression ratios were significantly elevated by phenytoin in the sensory cortex [S1BF: *t*_(10)_ = 2.783, *P* = 0.019] and amygdala [BLA: *t*_(10)_ = 2.340, *P* = 0.041] (Figure 6A). The Kir4.1 expression in the Motor, PRh-Ect, BLA, and BMP also tended to increase, whereas the changes did not reach statistical significance. In addition, phenobarbital significantly increased the Kir4.1 expression ratios in the amygdala [MePD: *t*_(10)_ = 3.492, *P* = 0.006 and BLA: *t*_(10)_ = 2.434, *P* = 0.035] (Figure 6B). In contrast, treatment with ethosuximide (100 mg/kg, i.p. for 10 days) failed to affect the Kir4.1 expression in any brain regions examined (Figure 6C).

## Discussion

Evidence is accumulating that the dysfunction (reduced function or expression) of astrocytic Kir4.1 channels causes epileptic disorders, including not only EAST/SeSAME syndrome with *KCNJ10* mutations (Bockenhauer et al., 2009; Scholl et al., 2009; Reichold et al., 2010), but also idiopathic epilepsy (Das et al., 2012; Heuser et al., 2012; Steinhäuser et al., 2012). These findings suggest that enhancement of Kir4.1 channel activities can prevent the development of epilepsy (epileptogenesis) by facilitating astrocytic spatial potassium buffering. The present study demonstrated for the first time that several antiepileptic drugs, which are commonly effective for GTCSs in patients, enhance the astrocytic Kir4.1 expression in the limbic regions. Valproate significantly elevated the astrocytic Kir4.1 expression in the amygdala, hippocampus and cerebral cortex, in a dose-and time-dependent manner. Phenytoin and phenobarbital also increased the Kir4.1 expression in the amygdala region. In addition, up-regulation of Kir4.1 expression by these agents did not accompany the increase in the number of astrocytes (astrogliosis). Limbic structures such as the amygdala have been generally recognized as sites closely related to epileptogenesis in animal models of epilepsy (McNamara, 1984; Morimoto et al., 2004). Moreover, human limbic regions are also involved in seizure generation not only in temporal lobe epilepsy, the most common type of adult localization-related epilepsy, but also in epilepsy induced by autoimmune encephalitis (Tatum, 2012; Melzer et al., 2015). Thus, our results suggest that the elevation of astrocytic Kir4.1 expression in limbic regions by the antiepileptic drugs contributes to their antiepileptic actions. Indeed, in our preliminary studies using audiogenic seizure susceptible *Lgi1*^*L*385*R*^ mutant rats (Baulac et al., 2012; Fumoto et al., 2014), repeated treatment with valproate alleviated epileptogenesis (development of seizure susceptibility) of the *Lgi1*^*L*385*R*^ mutant rats which exhibited down-regulation of astrocytic Kir4.1 expression (Kinboshi et al., 2017b).

Valproate inhibits GABA transaminase and increases GABA levels, thereby enhancing inhibitory GABAergic activities (Vajda and Eadie, 2014). Phenobarbital also activates the GABAergic system by prolonging the opening time of chloride ion channels within GABA_A_ receptors. In addition, both valproate and phenytoin possess an inhibitory action against voltage-gated Na^+^ channels. All these actions of antiepileptic drugs reduce neural excitability and contribute to an acute inhibitory action on seizure induction. Besides the acute actions, repeated treatments with these antiepileptics are known to exert, to some extent, prophylactic effects in chronic epilepsy, although such usage are sometimes limited by their side effects and/or drug interactions (e.g., enzyme-inducing properties) (Iudice and Murri, 2000; Michelucci, 2006; Torbic et al., 2013). Indeed, valproate reportedly had the potential to prevent epileptogenesis although the underlying mechanisms remain unclear (Silver et al., 1991; Bolanos et al., 1998; Hashimoto et al., 2003). The present fact that the up-regulation of Kir4.1 channels by antiepileptics was mostly manifested after repeated treatments suggests that the elevated expression of Kir4.1 channels may contribute to the seizure-preventive (prophylactic) actions of these agents.

Ethosuximide specifically alleviates absence seizures and does not affect (or sometimes worsen) GTCSs. It inhibits the low threshold T-type Ca^2+^ currents in thalamic neurons, although other mechanisms (e.g., inhibition of the non-inactivating Na^+^ currents and the Ca^2+^-activated K^+^ currents) are also proposed (Crunelli and Leresche, 2002). Interestingly, ethosuximide failed to affect Kir4.1 expression in any brain regions examined. Therefore, Kir4.1 channels may not be involved in preventive effects of ethosuximide on absence seizures. This is consistent with our previous findings that down-regulation of Kir4.1 expression was observed only in the GTCSs model (e.g., Noda epileptic rats), but not in the absence seizure model (Groggy rats), implying that pathophysiological alterations of Kir4.1 are not linked to non-convulsive absence seizures (Harada et al., 2013, 2014; Ohno et al., 2015).

In conclusion, we evaluated the effects of the antiepileptic drugs, valproate, phenytoin, phenobarbital and ethosuximide, on expressional levels of astrocytic Kir4.1 channels in rats. Valproate, phenytoin and phenobarbital, which commonly alleviate GTCSs, significantly increased Kir4.1 expression in the limbic regions (e.g., amygdala) without affecting the number of astrocytes. Up-regulation of Kir4.1 channels by valproate occurred in a dose- and treatment period-dependent manner. In contrast, treatment of rats with ethosuximide, which selectively ameliorates absence seizures, did not affect Kir4.1 expression. The present results demonstrated for the first time that antiepileptics (e.g., valproate, phenytoin and phenobarbital) up-regulate astrocytic Kir4.1 channels in the amygdala, which may contribute to their clinical efficacy in chronic epilepsy. However, it remains uncertain how these antiepileptics elevated the expression of Kir4.1 channels region-specifically in the limbic regions. Further studies are required to clarify the mechanisms underlying the control of astrocytic Kir4.1 expression by antiepileptic drugs.

## Author contributions

YO designed the research. TM, MK, YN, SS, AsO, YS, AoO, MF, and YO performed experiments. TM, MK, SS, HI, AI, and YO analyzed data. TM, MK, SS, HI, AI, and YO wrote the paper.

### Conflict of interest statement

The authors declare that the research was conducted in the absence of any commercial or financial relationships that could be construed as a potential conflict of interest.
